# Effects of Infection on Honey Bee Population Dynamics: A Model

**DOI:** 10.1371/journal.pone.0110237

**Published:** 2014-10-16

**Authors:** Matt I. Betti, Lindi M. Wahl, Mair Zamir

**Affiliations:** 1 Department of Applied Mathematics, Western University, London, Ontario, Canada; 2 Department of Medical Biophysics, Western University, London, Ontario, Canada; University of North Carolina, Greensboro, United States of America

## Abstract

We propose a model that combines the dynamics of the spread of disease within a bee colony with the underlying demographic dynamics of the colony to determine the ultimate fate of the colony under different scenarios. The model suggests that key factors in the survival or collapse of a honey bee colony in the face of an infection are the rate of transmission of the infection and the disease-induced death rate. An increase in the disease-induced death rate, which can be thought of as an increase in the severity of the disease, may actually help the colony overcome the disease and survive through winter. By contrast, an increase in the transmission rate, which means that bees are being infected at an earlier age, has a drastic deleterious effect. Another important finding relates to the timing of infection in relation to the onset of winter, indicating that in a time interval of approximately 20 days before the onset of winter the colony is most affected by the onset of infection. The results suggest further that the age of recruitment of hive bees to foraging duties is a good early marker for the survival or collapse of a honey bee colony in the face of infection, which is consistent with experimental evidence but the model provides insight into the underlying mechanisms. The most important result of the study is a clear distinction between an exposure of the honey bee colony to an environmental hazard such as pesticides or insecticides, or an exposure to an infectious disease. The results indicate unequivocally that in the scenarios that we have examined, and perhaps more generally, an infectious disease is far more hazardous to the survival of a bee colony than an environmental hazard that causes an equal death rate in foraging bees.

## Introduction

The widespread collapse of honey bee colonies has been the subject of much discussion and research in recent years [1]–[3]. Aside from their ecological importance [4], honey bee populations have a large economical impact on agriculture in North America, Europe, the Middle East, and Japan [5]–[7].

The focus of research has been largely on environmental factors outside the hive, such as pesticides or insecticides, which may cause death or injury to foraging bees and jeopardize their return to the hive. The reduced number of foraging bees then leads to younger hive bees being recruited prematurely to perform foraging duties and this chain reaction ultimately leads to a disruption in the dynamics of the colony as a whole. Examples of this scenario would be produced by the effects of various pesticides to which foraging bees are exposed in the course of their duties [2], [ 8]. Other factors in the same category include possible disruptions to the bees' navigation system by mobile phones or other electronic devices, again to the effect of jeopardizing their return to the hive and thereby reducing their numbers [9].

A key element in this category of disruption to honey bee population dynamics is the untimely *death* of a certain proportion of foraging bees outside the hive and the consequences of this on the colony as a whole. An important question here concerns the threshold in the death rate of foraging bees that would determine the survival or collapse of the bee colony. This was examined recently in two papers by Khoury et al. [10], [ 11].

In the present paper we consider a different category of disruption to the healthy dynamics of a bee colony, namely one in which the key hazard is an *infection* by a communicable disease acquired by foraging bees outside the hive. The key difference here is that foraging bees that have been infected would then transport the disease into the hive and go on to infect other members of the colony *within the hive*. Here too the affected bees will ultimately suffer an untimely death, but the effects on the dynamics of the colony are clearly more complex because the infection in this case may now involve all members of the colony. We sought a model that would allow a comparison between the effects of these two categories of hazards (pesticide versus infection) on the ultimate fate of the bee colony.

Disease in honey bee colonies has been studied previously by Sumpter et al. [12] who modeled the effects of Varroa mites on the brood and on the adult worker bees. The focus of the model was on the relationship between the mite population within a hive and its role in virus transmission within the hive. A study by Ratti et al. [13] examined the transmission of viruses via Varroa mites, using an SIR-framework with the mites as vectors for transmission.

In the present paper we propose a more general model which combines the normal dynamics of a honey bee colony with the dynamics of an infectious disease which is acquired outside the hive but ultimately spreads to the rest of the colony. As a working example, we use a disease known as “Nosema” which is a common disease affecting both hive bees and foraging bees [14]. Nosema is caused by a microsporidian parasite with two common strains: *Nosema ceranae* and *Nosema apis*. The former was first discovered in Asian honey bees (*Apis ceranae*) and the latter is common among European honey bees (*Apis mellifera*). A key factor in the collapse of honey bee colonies in recent years is thought to be the introduction of *Nosema ceranae* to *Apis mellifera* [15].

The main aim of the model is to provide a general tool for determining the ultimate fate of a honey bee colony under this fairly common hazard. In particular, we identify key variables that determine the collapse or survival of the bee colony, namely the severity of the disease and the rate of transmission, and examine different scenarios using different combinations of these variables. Winter is an important phase in the normal demographic dynamics of a bee colony; the queen lays fewer eggs and foraging bees return to and remain within the hive [16], [ 17]. Therefore, the time interval between the onset of disease and the onset of winter may play a critical role in the ultimate survival or collapse of the colony in the face of an infection. We show that the model can be used to explore potential markers of the presence of the disease within the bee colony and of the ultimate fate of the colony under different scenarios.

## Background

### 2.1 Normal Demographics of a Honey Bee Colony

Honey bee colonies are complex societies in which different members of the colony have specialized functions that serve the entire colony, thus making members of the colony highly dependent on each other.

The queen can live up to three years, is responsible for laying eggs, and during peak season may lay up to 2000 eggs per day [18]. In this function the queen is dependent on worker bees [19]. The worker bees emerge from fertilized eggs of the queen and consist of females who maintain the hive and gather resources, and males who mate with the queen to produce more eggs [20]. Drones are born from unfertilized eggs of the queen [20] and typically making up less than 5% of the hive population [20], [ 21]. Because they do not contribute to the colony work force, and because of their small numbers, they are generally neglected when considering the dynamics of the colony as a whole.

Female hive bees, following a transition period, leave the hive to start foraging duties and usually forage until their death. The age at which they start foraging duties is variable, depending on the state of the colony and its needs. If the number of forager bees is lower than is required for meeting the colony needs, hive bees will begin foraging duties at a younger age [22]. If the number of forager bees is higher than required, behavioural maturation of hive bees will be regulated by a pheromone, ethyl oleate, produced by the foragers. This process is usually referred to as “social inhibition” [23]. Similarly, if the number of hive bees is too low, it is possible for foragers to revert back to hive bee duties [22].

As the temperature drops outside the hive, foraging becomes less frequent, the queen begins to lay fewer eggs [19], and drones are expelled from the hive to save hive resources [20]. When the temperature drops below a certain threshold, the colony enters a winter phase in which the queen will cease to lay eggs [16] and any remaining foraging bees will return to the hive. During winter the entire hive population surrounds the queen in order to maintain a temperature of 34–36°C within the hive [20].

### 2.2 Nosema Infection

Nosema, also known as “Nosemosis”, is an infection affecting honey bees that is spread by the microsporidian parasites in the Nosema family. *Nosema ceranae* is of particular interest, as it is thought to be linked to colony collapse incidents [15], [ 24]. We use this disease only as an example to illustrate the utility of the model. The choice was motivated by the availability of parameter values which allowed us to examine some realistic scenarios of the dynamics of the bee colony in the presence of infection.

Within the bee colony, Nosema is typically spread via fecal-oral transmission. Adult bees will contract Nosema either from eating food contaminated by infected bees, or while ridding the hive of infected fecal matter [25]. There is also evidence that Nosema can be spread via oral-oral transmission, through feeding [26].

While it is typically asymptomatic at the level of individual bees, Nosema has some symptoms that can be observed at the colony level [14], [ 27]. Stevanovic et al. [27] observed in 2013 that colonies infected by the parasite *Nosema ceranae* exhibited many of the classic signs that precede colony collapse.

Much of the experimental research linking Nosema infection to colony collapse is based on correlated observations, but direct cause and effect evidence is lacking [14]. Our model aims to provide a possible mechanism for this linkage in terms of the interplay between the dynamics of the infection and the normal dynamics of the honey bee colony.

### Mathematical Model

In what follows we present a mathematical model that combines the normal demographic dynamics of a honey bee colony with the dynamics of an infection affecting foraging bees outside the hive at first and then spreading to the rest of the colony. We follow a model for the basic dynamics of a bee colony in the absence of disease presented recently by Khoury et al. [10], [ 11], in which the adult bee population is divided into a number of hive bees *H*, and a number of foraging bees *F*. In the model to be described below we extend this division into four categories, namely susceptible hive bees *H_S_*, infected hive bees *H_I_*, susceptible foraging bees *F_S_*, and infected foraging bees *F_I_*. Equations governing each of these four populations during the active and winter seasons are presented in the following section.

### 3.1 Governing Equations: Active Season

The rate of change in time *t* (days) of the susceptible hive bee population *H_S_* during the active season is assumed to be governed by
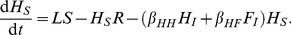
(1)


In the first term on the right *L* is the queen's egg laying rate per day and *S* is the proportion of those eggs that survive both larval and pupal stages to yield mature bees. This proportion is a function of the total number of hive bees and of the amount of food *f* available within the hive because the brood requires food as well as a sufficient number of supporting hive bees in order to survive [28]. Following [11] we take
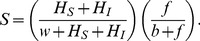
(2)


This function is constructed such that the value of *S* saturates at 1.0 in the limiting case when the amount of food *f* and the total number of hive bees *H_S_*+*H_I_* are sufficiently large to ensure the survival of 100% of the eggs laid by the queen. The parameters *b* and *w* determine at what values of *f* and *H_S_*+*H_I_* this saturation occurs and they will be discussed later.

In the second term on the right of Eq. 1, *R* is the proportion of maturing hive bees *H_S_* that are being recruited to foraging duties. As discussed earlier, and following [11], we assume that recruitment is increased when either food stores or forager populations are low and recruitment is reduced when food stores and forager populations are in excess. Note that in an overabundance of foragers, *R* may become negative, which implies that foragers are reverting to hive duties.
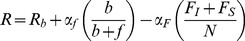
(3)where *R_b_* is the baseline recruitment rate in the absence of foragers but sufficient food stores, *α_f_* is a weighting of the effect of low food, *α_F_* is a weighting of the effect of excess foragers on recruitment, and *N* = *F_I_*+*F_S_*+*H_S_*+*H_I_* is the colony adult population size. The Average Age of Recruitment to Foraging (AARF) at any point in time is equal to 1/*R*.

The last term in Eq. 1 determines the rate at which susceptible hive bees become infected. The transmission rate per day per susceptible hive bee is given by (*β_HH_H_I_*+*β_HF_F_I_*), where *β_HH_* is the contact rate between hive bees and *β_HF_* is that between hive bees and foraging bees.

Hive bees are safe within the hive environment under normal circumstances, surviving up to 6 months over winter [10], [ 20]. It is therefore assumed that the natural death rate of hive bees is negligible compared to their recruitment rate to foraging duties.

For the rate of change of the infected hive bee population, we take
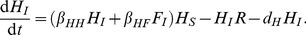
(4)


Infected hive bees continue to be recruited to foraging duties but, unlike their healthy counterparts, they are at risk of dying from the disease before they do so; *d_H_* is the rate at which this occurs.

Susceptible foragers are recruited from susceptible hive bees and may subsequently suffer natural death, at a rate *m*, or become infected. Their rate of change is therefore governed by
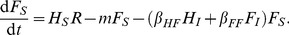
(5)


Infected foragers are recruited from infected hive bees or are susceptible foragers that have become infected. If the death rate from the infection is assumed to be *d_F_* then their rate of change is governed by
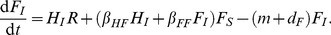
(6)


Food is brought into the hive by foragers, either healthy or infected. Although infected foragers may forage less efficiently, for simplicity we assume the same foraging rate, *c* (gm/day) per forager. The collected food is then consumed by both foragers and hive bees and for simplicity again we assume the same consumption rate, 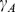 (gm/day). The amount of food consumed by the larvae is substantial. We assume that the number of larvae is proportional to the number of surviving eggs and that the larvae consume food at a rate of 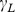 (gm/day). The amount of food available at time *t* is thus given by
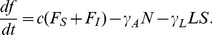
(7)


**Figure 1:**
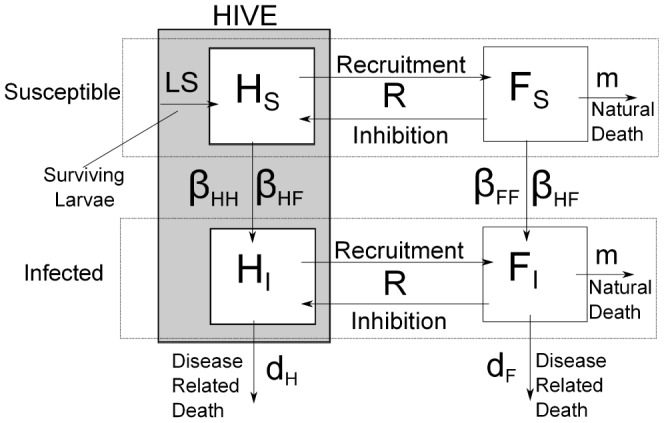
A compartmental diagram of the dynamics of the honey bee colony combined with the dynamics of an infectious disease. The susceptible and infected hive bees, *H_S_* and *H_I_* live within the hive. New susceptible hive bees are generated by surviving brood through the survival function, *S*. New infected hive bees are generated through interactions of susceptible hive bees with infected hive bees and infected foragers at rates *β_HH_* and *β_HF_*. Hive bees are recruited to foraging duties through the recruitment function *R*, which also allows for the reversal of duties, from foraging to hive duties. Foragers move into the infected compartment via interactions with infected hive bees and infected foragers at rates *β_HF_* and *β_FF_*. All infected bees die at rates *d_H_* or *d_F_*, and foragers die naturally at rate *m*.

The full dynamics of the bee colony are thus governed by Eqs. 1, 4, 5, 6 and 7 to be solved simultaneously. A compartmental diagram of these dynamics is shown in Figure 1.

### 3.2 Governing Equations: Winter

During winter the rate of egg laying by the queen is considerably diminished, and in harsh climates the queen may cease laying eggs completely [16]. For simplicity, in our model simulations therefore we take *L* = 0 for the winter season.

Foraging resources become scarce in winter and foraging bees return to the hive to join hive bees in their effort to keep the hive warm [16]. The two groups thus perform the same duties in winter and there is no longer any recruitment from hive to foraging duties. We therefore set *R* = 0, although we maintain the separate identities of the two groups in the model in order to track the behaviors of bees that were foraging before winter against those that were hive bees.

Since there is no foraging in winter, food production halts and we set *c* = 0. Also, bees are able to survive longer in winter than they do outside the hive during the active season [29]. Thus the new natural death rate for both hive bees and foraging bees during the winter season is set to be *m_W_*.

Introducing these changes into the equations governing the dynamics of the colony (Eqs. 1, 4, 5, 6, 7) we obtain the corresponding equations for the winter season:
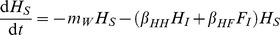
(8)

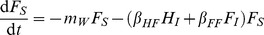
(9)

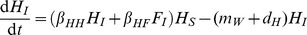
(10)

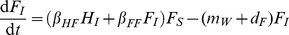
(11)

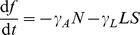
(12)


### 3.3 Parameter Values

The model presented in Section 3 contains a total of 13 parameters. Of these, 10 parameters relate to the baseline demographic dynamics of a honey bee colony, in the absence of disease, for which empirical estimates are available in the literature. In particular, we consider a bee colony in which the maximum rate (*L* in Eq. 2) of egg laying by the queen is 2000 eggs/day and take *w* = 5000 [10]. Hive bees spend, on average, a minimum of 4 days in the hive before being recruited to foraging duties [30], and foragers will not revert to hive duties unless one-third of the bee population is foraging [10]. Based on these values, and following [10], we take *R_b_* = 0.25 and *α_f_* = 0.75. In the complete absence of food, recruitment of foragers will double [31], thus we take *α_F_* = 0.25. Foraging bees are estimated to live approximately 6.76 days outside the hive [32], thus we set *m* = 0.14 deaths per bee per day.

The parameter *b* in Eq. 2 is the amount of food required to ensure the survival of half of the eggs to maturation. Based on the observation that the effects of low food stores become evident when there is less than 1 kg of stored food [10], we take *b* = 500. It is estimated that as long as the hive is in an environment that provides sufficient food resources, a forager will return with *c* = 0.1 g of food per day [33], [ 34]. It is also estimated that the daily food requirement of each member of the brood is 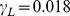 g and that of an adult hive or foraging bee is 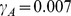 g [10], [ 11], [ 34].

We assume that both the rate of food consumption and the transmission rate of the disease remain the same during the active and winter seasons. However, empirical evidence indicates that bees live longer in winter, surviving up to six months [29], and on that basis we take the natural death rate in winter, *m_W_* = 1/180 deaths per bee per day.

The remaining parameters relate to the dynamics of the disease and, as stated earlier, we have chosen *Nosema ceranae* particularly because of the availability of parameter values. The effect of *Nosema ceranae* infection is estimated to double the mortality rate of adult bees [35]. On that basis we take *d_H_* = *d_F_* = *m* = 0.14 deaths per bee per day. For the rates of transmission at first we considered different values of *β_HH_*, *β_HF_*, *β_FF_*. Following some preliminary simulations, however, we found these different values have only a marginal qualitative effect on the overall dynamics of the disease. Accordingly, and in the absence of any field values on which to base a meaningful examination of this issue, the simulations which we present in this paper are based on taking *β_HH_* = *β_HF_* = *β_FF_* = *β*. Generally, transmission of the disease is mediated via the food stores [26], which makes it difficult in practice to measure the rate of transmission from an infected bee to a susceptible bee.

A summary of all the parameter values we used is provided in Table 1.

**Table 1:** Parameter values and references.

*L*	maximum rate of egg laying	2000 eggs/day	[10]
*W*	number of hive bees for 50% egg survival	5000 bees	[10]
*R_b_*	baseline recruitment rate	25%/day	[30]
*α_f_*	maximum additional recruitment in absence of food	25%/day	[31]
*α_F_*	effect of excess foragers on recruitment	75%/day	[10]
*m*	natural death rate of foragers (active season)	14%/day	[32]
*m_w_*	natural death rate of foragers and hive bees (winter)	0.56%/day	[29]
*b*	mass of food stored for 50% egg survival	500 g	[11]
*c*	food gathered per day per forager	0.1 g/day	[33]
*γ*	daily food requirement per adult bee	0.007 g	[11]
*d_H_*	death rate of hive bees due to infection	14%/day	[35]
*d_F_*	death rate of foragers due to infection	14%/day	[35]
*β_HH_*	disease transmission rate: hive bee to hive bee	variable	
*β_HF_*	disease transmission rate: hive bee to forager	variable	
*β_FH_*	disease transmission rate: forager to hive bee	variable	
*β_FF_*	disease transmission rate: forager to forager	variable	

## Results

In what follows we present the results of numerical simulations of key scenarios that illustrate the main dynamics of the bee colony in the presence of disease.

To simulate the dynamics of the bee colony, we integrate the governing equations (Eqs. 1, 4, 5, 6, 7) numerically, with initial conditions *H_I_*(0) = *F_I_*(0) = 0 and *H_S_*(0), *F_S_*(0) based on steady state values for the disease free equilibrium which can be determined analytically. The food stores, *f*, continue to grow throughout the active season, and we have found that the results are not sensitive to the initial value of food in the hive. We present scenarios in which the dynamics of the disease begin at day 100. The initial onset of infection is simulated by turning 10% of the susceptible foragers into infected foragers.

### Scenario 0

**Figure 2:**
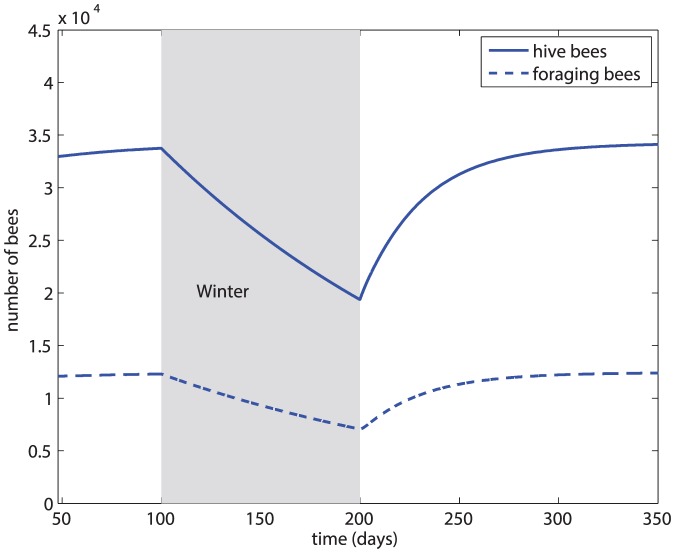
Baseline demographic dynamics of the honey bee colony in the absence of disease.

In this scenario we illustrate the baseline demographic dynamics of the colony in the absence of disease, particularly to highlight the natural seasonal variations. Thus, for this purpose, in this case we introduce winter after the initial 100 days of integration. The results are shown in Figure 2. The figure shows that both the hive and the foraging bee populations decrease (from natural death) over winter, but sufficient numbers remain (because of a lower death rate within the safety of the hive) after a fairly long winter of 100 days. At day 200, the active season resumes and the colony rebounds to the pre-winter equilibrium.

### Scenario 1

**Figure 3:**
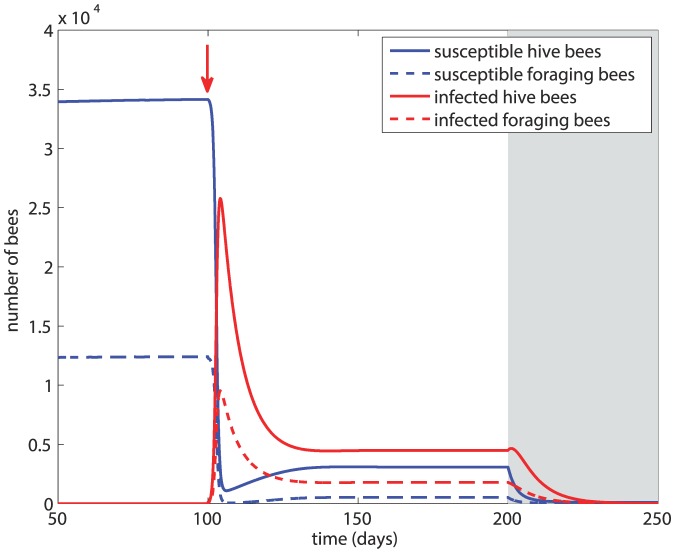
Scenario 1: Colony dynamics in the presence of disease with *β* = 5×10^−5^, *d_H_* = *d_F_* = 0.14. Red arrow  =  onset of infection, grey shading  =  winter.

In this scenario, after the initial 100 days we introduce infected foragers into the system, followed by winter 100 days later. The results are shown in Figure 3 based on *β* = 5×10^−5^ and *d_H_* = *d_F_* = *m* = 0.14. The figure shows that within about 5 days the susceptible bee population suffers a drastic drop and the majority of the hive bees have become infected. The infection greatly reduces the overall size of the colony but a new equilibrium is reached, with about 65% of the total population sustaining the infection. At the onset of winter, the size of the colony is not sustainable and within 50 days of winter the colony has collapsed.

### Scenario 2

**Figure 4:**
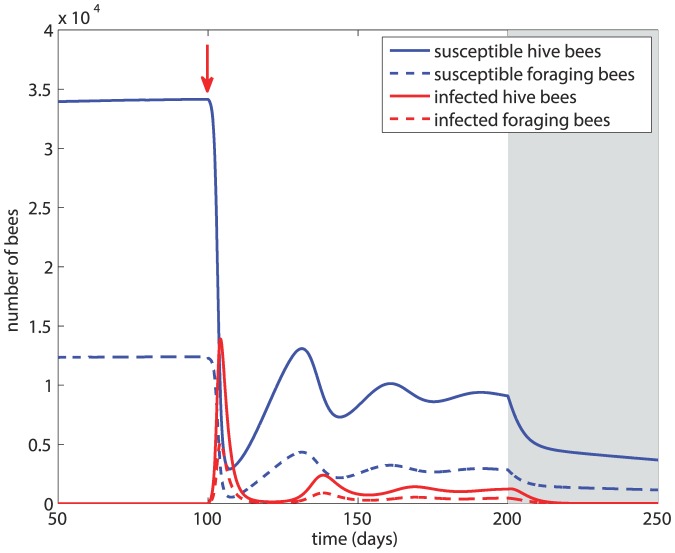
Scenario 2: Colony dynamics under a more severe infection represented by a higher death rates from the disease, with *β* = 5×10^−5^, *d_H_* = *d_F_* = 0.56. Red arrow  =  onset of infection, grey shading  =  winter.

In this scenario we examine the effect of a more severe infection in which the transmission rate is unchanged but the mortality rates from the disease are increased to *d_H_* = *d_F_* = 4*m* = 0.56. The results, in Figure 4, show that after an initial drastic drop, the population of susceptible bees begins to recover approximately 10 days after the onset of the infection. The small numbers of infected hive and forager bees lead to their quick demise soon after the onset of winter, and the disease is eradicated from the hive within 25 days of the onset of winter. Thus, in this case while the colony has sustained heavy losses from the infection, it survives winter with a viable number of bees and no disease. A more severe infection, in the sense that it kills faster, can therefore lead to the survival of the colony as a whole.

### Scenario 3

**Figure 5:**
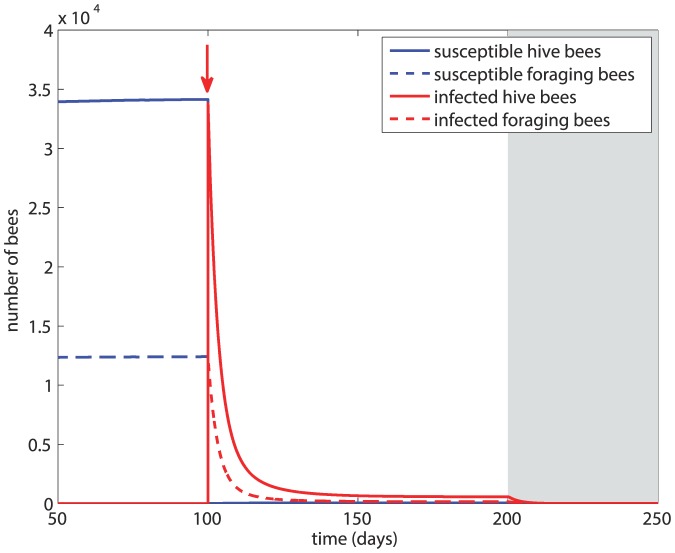
Scenario 3: Colony dynamics under a higher rate of transmission of the disease, with *β* = 5×10^−3^, *d_H_* = *d_F_* = 0.56. Red arrow  =  onset of infection, grey shading  =  winter.

In this scenario we examine the effect of an increased rate of transmission, setting *β* = 5×10^−3^ and *d_H_* = *d_F_* = 2*m* = 0.28. The results are shown in Figure 5. The infection spreads quickly through the colony, the susceptible population is almost immediately eradicated, and within 30 days the colony drops drastically to <10% of its size before infection. Thereafter, the colony population continues to dwindle slowly, and at the onset of winter it collapses within 10 days. For comparison, with the same natural death rate but in the absence of infection, the colony survives through winter and rebounds to its pre-winter level at the onset of the next active season as seen in Figure 2.

### Age of Recruitment to Foraging Duties

**Figure 6:**
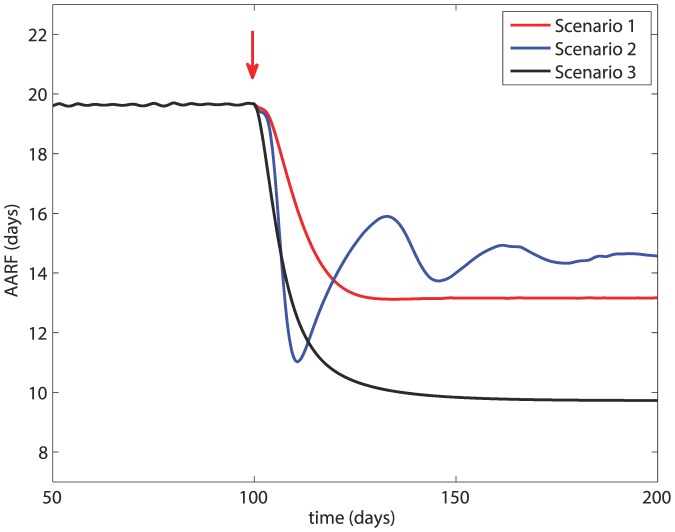
Average age of recruitment to foraging duties (AARF) under the three scenarios in Figures 3, 4, 5. Red arrow  =  onset of infection.

The average age at which hive bees are recruited to foraging duties (AARF) under the three scenarios is shown in Figure 6. The figure shows that AARF is an important marker of the health of the colony in the sense that a colony with a younger workforce can be taken as a sign of disease within the colony. In Scenario 1, AARF is reduced from 19.6 days before the onset of infection to 13.16 after the infection. In Scenario 2, with a higher disease-induced death rate, AARF is reduced to about 14.6 days, though fluctuating between 10 days and 16 days at first. In Scenario 3, with a higher rate of transmission of the infection, AARF is reduced drastically to 9.7 days.

**Figure 7:**
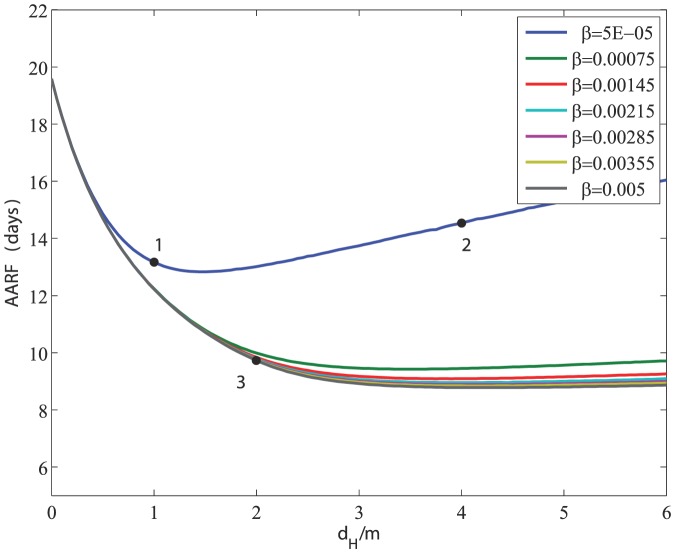
Relationship between the rate of transmission *β* and disease-induced death rates *d_H_* = *d_F_* in their effects on the Average Age of Recruitment to Foraging. The figure shows the effects of an increase of *β* and *d_H_* on the AARF. Note that the AARF becomes less sensitive to changes in *β* as *β* is increased. Meanwhile, for small *β*, an increase in *d_H_* can have a favourable effect on the AARF.

Figure 7 shows the complex relationship between the rate of transmission *β* and disease-induced the death rates *d_H_*, *d_F_* in their effects on the AARF. The figure shows that a combination of small *β* and large *d_H_* is favorable in that it leads to a higher value of the AARF. At higher values of *β*, however, the AARF becomes less sensitive to the value of *β* (as indicated by the clumping of the curves in that region). The position of the three scenarios in this relationship as shown in the figure, and their ultimate fate as described earlier, shows again that the AARF is an early marker of colony collapse, which has been supported by experimental evidence [36].

### Scenario 4

**Figure 8:**
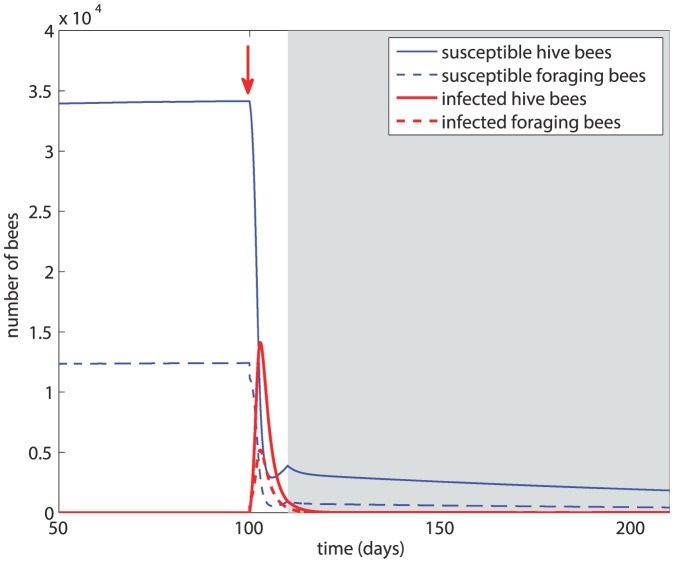
Scenario 4: *β* = 5×10^−5^, *d_H_* = *d_F_* = 0.56. Effect of the proximity of the onset of infection to the onset of winter. Red arrow  =  onset of infection, grey shading  =  winter.

In this scenario we examine the effect of the timing of the infection in relation to the onset of winter. Figure 8 shows the effect of infection occurring only 10 days before the onset of winter, compared with 100 days in earlier scenarios. The results, compared with those in Scenario 2, show that the disease is eradicated sooner by early winter. This is clearly because healthy bees live longer in the safety of the hive in winter, while the death rate from infection is unchanged.

**Figure 9:**
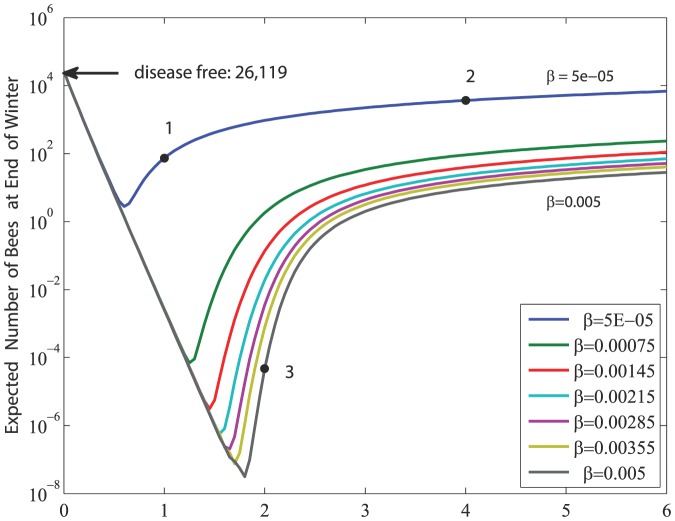
The expected size of the bee population at the end of winter as influenced by the severity of the disease (*d_H_* = *d_F_*) and the transmission rate of the disease (*β*). For comparison, the black arrow indicates the population size at end of winter in the absence of disease (Figure 2). The figure illustrates the different sensitivity to *β* and *d_H_*. Note that *d_H_* has a favourable effect for small *β* and *d_H_* large enough.

**Figure 10:**
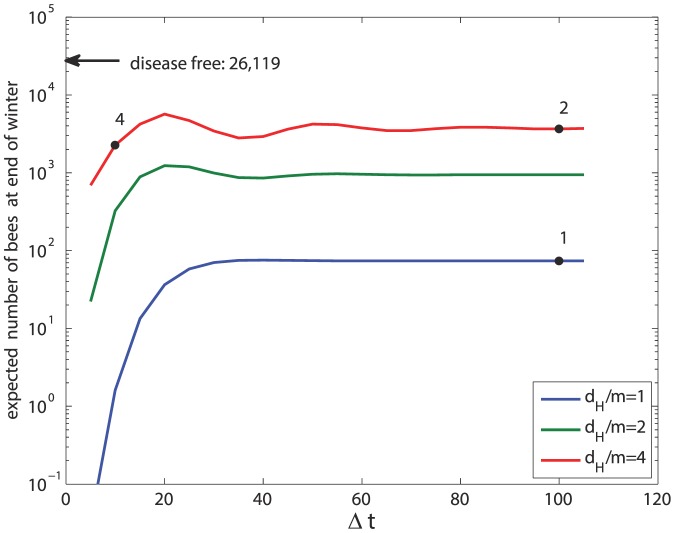
The expected size of the bee population at the end of winter as influenced by the time interval between the onset of infection and the beginning of winter (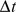), with *β* = 5×10^−5^. For comparison, the black arrow indicates the population size at end of winter in the absence of disease (Figure 2).

Another important indicator of the ultimate fate of the bee colony is the size of the bee population at the end of winter. While under all scenarios winter is taken to last 100 days, the size of the bee population at the end of winter is influenced by the severity of the disease (*d_H_*, *d_F_*), the transmission rate (*β*), and the time interval between the onset of infection and the beginning of winter which we shall denote 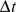. This complex relationship is shown first in Figure 9 for Scenarios 1, 2, 3 where 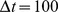 days in all three cases. Again, we see a decrease in sensitivity to *β* at higher values of *β*. Furthermore, an increase in the value of *d_H_* initially has an unfavorable effect on the colony size at end of winter, but at high values of *d_H_* this effect is reversed. The region of fractional values is included in Figure 9 only for (mathematical) completeness of the figure. Biologically, the region represents colonies that do not survive. By comparison, in Scenario 4 where 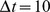 the size of the bee population at end of winter is reduced by 38% from that in Scenario 2 where the values of other parameters are the same. A more general indication of the dependence of the size of the bee population at end of winter on 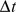 is shown in Figure 10. The figure shows that for 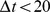 days or so, there is very high sensitivity to the value of 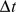, but for 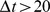 days or so this sensitivity is considerably diminished. This indicates that in the three weeks or so before winter the bee colony is most vulnerable to the risk of infection.

**Figure 11:**
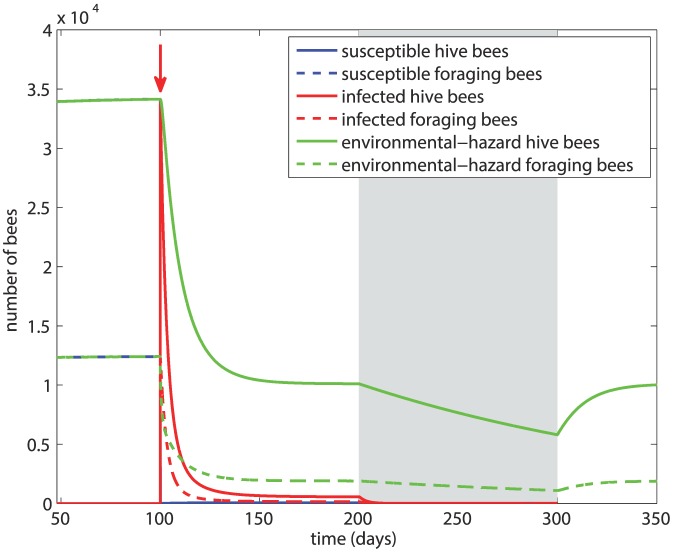
The stark difference between the dynamics of Scenario 3 with an environmental hazard scenario in which the death rate is increased (by the effects of pesticides, for example) *to equal the total death rate in Scenario 3*. The survival of the colony is almost guaranteed in the environmental hazard scenario while the collapse of the colony is almost guaranteed in the disease scenario.

Finally, in Figure 11 we compare the effects of two major types of hazards faced by a honey bee colony, one in which there is a simple increase in the death rate of foragers because of exposure to an environmental hazard and another in which the bees are exposed to an infectious disease. Specifically, in this figure we contrast the dynamics of Scenario 3 with the dynamics of an environmental hazard scenario in which the hive is disease-free but the death rate from the environmental hazard is *the same as the total death rate in Scenario* 3. Specifically, in Scenario 3 we had *d_F_* = 0.28, *d_H_* = 0.28, *m* = 0.14 for a total death rate of 0.7, thus, for a comparable environmental hazard scenario we take *m* = 0.7 and *d_F_* = *d_H_* = 0. The figure shows clearly that the survival of the colony is almost guaranteed in the environmental hazard scenario, while the collapse of the colony is almost guaranteed in the disease scenario.

**Figure 12:**
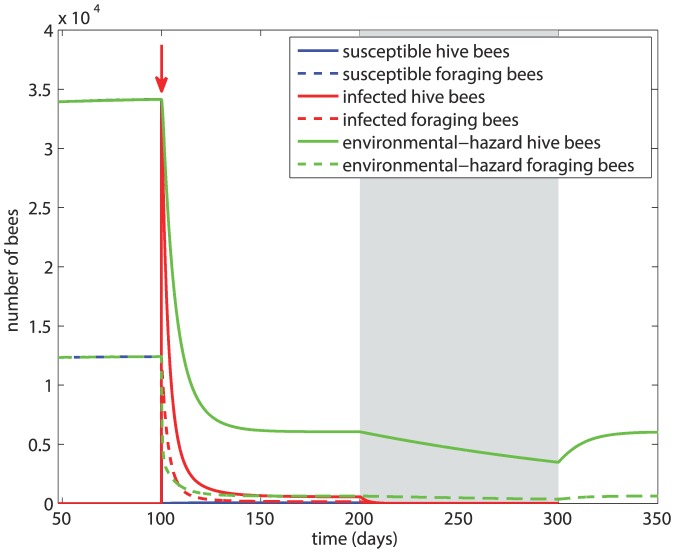
An alternative comparison of the dynamics of Scenario 3 with an environmental hazard scenario in which the comparison between the two hazards is based not on the total death rate as in Figure 11 but on the average lifespan of bees being the same in both cases.

This comparison is clearly approximate because the three components of death rate in the infectious disease case (*d_F_*, *d_H_*, *m*) are independent of each other and therefore their sum is not accurately comparable to the total death rate in the environmental hazard case. For this reason, in Figure 12 we consider another comparison in which the dynamics of the two hazards are such that the *average lifespan of bees is the same in both cases*. The results again show that the colony survives under the environmental hazard.

## Discussions and Conclusions

The main aim of this study was to construct a model for examining the way in which the dynamics of a honey bee colony are affected by an infection. We present this model in terms of a set of governing equations representing the interplay between the dynamics of the spread of the disease and the demographic dynamics of the bee colony. Up to this point the model is fairly general in regard to the specific characteristics of the colony or the disease and can thus be adapted to a variety of specific cases by an appropriate choice of parameter values. To illustrate the utility of the model, we chose parameter values associated with *Nosema ceranae* which has been well studied experimentally. Our findings, compared with those found experimentally are summarized in Table 2.

**Table 2:** Tabulated results from the model scenarios 1, 2, and 3 and experimental data from [36] and [35].

	Exp.	1	2	3
AARF- Healthy	14–21	19.6	19.6	19.6
AARF- Infected	7–16	13.1	14.6	9.7
(*H_I_*+*F_I_*)/*N*	45%	64%	12.2%	92%

The last row shows the percentage of the population infected at the endemic equilibrium, and the experimental value is the threshold value which leads to over-winter colony collapse.

The model suggests that key factors in the survival or collapse of a honey bee colony in the face of an infection are the rate of transmission of the infection *β* and the disease-induced death rates, *d_H_* and *d_F_*. An increase in the disease-induced death rates, which can be thought of as an increase in the severity of the disease, may actually help the colony overcome the disease and survive through winter (Scenario 2), which is consistent with SIR models of epidemics. By contrast, an increase in the transmission rate, which means that bees are being infected at an earlier age, has a drastic deleterious effect (Scenario 3).

Another important finding relates to the timing of infection in relation to the onset of winter. The results (Figure 10) suggest that in a time interval of approximately 20 days before the onset of winter the colony is most affected by the onset of infection. An infection during this “dangerous” time period is more likely to lead to colony collapse because the number of bees surviving through winter becomes unviable for a rebound of the colony in the new active season. Outside this dangerous time period, i.e. for 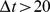 days, the survival of the colony is no longer critically affected by the timing of infection. It must be emphasized that the numerical value of 20 days for this dangerous time period is likely not a “universal” value but one that is specific to the choice of parameter values we used both for the colony and the disease. With other combinations of colony and disease parameters, the model can be used to find the corresponding critical time period.

Our results (Figures 6 and 7) suggest that the AARF is a good early marker for the survival or collapse of a honey bee colony in the face of infection. This is consistent with experimental evidence in [36] but the model and the results in Figures 6 and 7 provide an insight into the underlying mechanisms for this.

Finally, an important result of this study is the clear distinction between two major types of hazards faced by a honey bee colony, namely, one in which there is a simple increase in the death rate of bees because of exposure to an environmental hazard such as pesticides or insecticides, and another in which the bees are exposed to an infectious disease. The results in Figure 11 show that an exposure to an infectious disease is almost guaranteed to lead to colony collapse while under an environmental hazard the colony has a good chance of survival. This conclusion is confirmed by the results of Figure 12 in which the comparison between the two hazards is based not on the total death rate but on the average lifespan of bees being the same in both cases. Since an environmental hazard in the first place affects only forager bees, the comparison in this case is equivalent to considering a more severe environmental hazard than that in Figure 11, or to considering the long term consequences of an environmental hazard as it affects the demographics of the colony. Together, the two comparisons lead us to suspect that, under comparable death rates and the range of disease transmission rates which we have considered, an infectious disease may typically be more hazardous to the survival of a bee colony than an exposure to pesticide or insecticide.
